# Erythropoietin Protects Epithelial Cells from Excessive Autophagy and Apoptosis in Experimental Neonatal Necrotizing Enterocolitis

**DOI:** 10.1371/journal.pone.0069620

**Published:** 2013-07-25

**Authors:** Yueyue Yu, Sheng-Ru Shiou, Yuee Guo, Lei Lu, Maria Westerhoff, Jun Sun, Elaine O. Petrof, Erika C. Claud

**Affiliations:** 1 Department of Pediatrics, Section of Neonatology, University of Chicago, Chicago, Illinois, United States of America; 2 Department of Medicine, Section of Gastroenterology, University of Chicago, Chicago, Illinois, United States of America; 3 Department of Pathology, University of Washington, Seattle, Washington, United States of America; 4 Department of Medicine, Rush University, Chicago, Illinois, United States of America; 5 Department of Medicine, Queen’s University, Kingston, Ontario, Canada; Emory University School of Medicine, United States of America

## Abstract

Neonatal necrotizing enterocolitis (NEC) is a devastating gastrointestinal disease of preterm infants. Increased intestinal epithelium permeability is an early event in NEC pathogenesis. Autophagy and apoptosis are induced by multiple stress pathways which may impact the intestinal barrier, and they have been associated with pathogenesis of diverse gastrointestinal diseases including inflammatory bowel disease. Using both *in vitro* and *in vivo* models, this study investigates autophagy and apoptosis under experimental NEC stresses. Furthermore this study evaluates the effect of erythropoietin (Epo), a component of breast milk previously shown to decrease the incidence of NEC and to preserve intestinal barrier function, on intestinal autophagy and apoptosis. It was found that autophagy and apoptosis are both rapidly up regulated in NEC *in vivo* as indicated by increased expression of the autophagy markers Beclin 1 and LC3II, and by evidence of apoptosis by TUNEL and cleaved caspase-3 staining. In the rat NEC experimental model, autophagy preceded the onset of apoptosis in intestine. *In vitro* studies suggested that Epo supplementation significantly decreased both autophagy and apoptosis via the Akt/mTOR signaling pathway and the MAPK/ERK pathway respectively. These results suggest that Epo protects intestinal epithelium from excessive autophagy and apoptosis in experimental NEC.

## Introduction

Neonatal necrotizing enterocolitis (NEC) is a devastating gastrointestinal disease, and a leading cause of morbidity and mortality in premature infants. Prematurity, hypoxia, bacterial colonization, and enteral feeding have been cited as the major risk factors for NEC, however the etiology of NEC remains unclear [1]. Understanding the key triggers of the cascade of events leading to the intestinal injury in NEC is necessary to develop prevention and treatment strategies [2], [3]. Autophagy is an evolutionarily conserved homeostatic process which occurs in all cells at low basal levels for protein and organelle turnover. During nutrient starvation or trophic growth factor withdrawal, autophagy is up regulated to supply cells with metabolites as survival fuel. Therefore, autophagy is generally regarded as a cytoprotective process. However, under extended periods of stress, when massive autophagy exceeds a safe threshold, it can also kill the cells [4]–[6]. Impaired autophagy has been linked to the pathogenesis of diverse diseases including cancer, neurodegeneration, aging, tuberculosis, and inflammatory bowel disease (IBD). Polymorphisms of two autophagy related genes have been identified and strongly correlated with Crohn’s disease (CD) [7], [8]. Autophagy has also been reported to occur in the intestinal epithelium of neonatal piglets in early postnatal life [9], and to be activated in the intestinal epithelium of NEC patients and in the ileum of experimental NEC rats [10]. However, the connection between activation of autophagy and induction of NEC is still poorly understood.

Autophagy is a complicated cellular process, and genetic screens in yeast have identified over 30 distinct autophagy-related (Atg) genes, many of which have mammalian homologs [11]. The induction of autophagy under conditions of nutrient deprivation is tightly controlled by mammalian target of rapamycin (mTOR), a nutrient sensor and a negative regulator of autophagy. Upon receiving upstream signals, such as the 5′-AMP-activated protein kinase (AMPK), mTOR is inactivated which triggers the autophagy cascade [12]. During autophagy, a double membrane vesicle (autophagosome) forms, that sequesters the intracellular organelles and part of the cytoplasm. The autophagosome then fuses with the lysosome to form the autolysosome, exposing the inner compartment to lysosomal hydrolases for bulk degradation [13]. The formation of the autophagosome is mediated by the Atg12-Atg5-Atg16 complex and microtubule-associated protein light chain 3 (LC3I)-phospholipid conjugates (LC3II) [14], [15]. Lipid conjugation leads to the conversion of the soluble form of LC3I to the autophagosome-associated form LC3II. This lipidation and recruitment to the autophagosome results in a shift from diffuse to punctate staining, thus LC3II is used as a hallmark of autophagy. p62/SQSTM1 is a well known autophagy substrate. p62 incorporates into autophagosomes through interaction with LC3 and is efficiently degraded by autophagy. Inhibition of autophagy results in rapid accumulation of cellular p62, while decreased p62 levels are associated with autophagy activation, thus p62 is employed as an indicator of autophagy flux [16]. Beclin 1, which is a component of a type III PI3-kinase complex involved in the nucleation of the autophagic vesicle, is another marker of autophagy [17].

Autophagy is a survival strategy for host defense, whereas apoptosis is a programmed cell death. Autophagy and apoptosis are two interconnected self-destructive processes. Crosstalk between these two processes is suggested by common inducers, regulators and signaling pathways [5], [18]. Under homeostatic conditions, intestinal epithelial apoptosis predominantly occurs at the villus tip and is highly regulated without disturbing intestinal barrier function or evoking inflammation [19]. However, under pathological conditions, excessive apoptosis has been found along the entire length of the villi and in the crypts [20], [21]. In an experimental rat model of NEC, it has been revealed that intestinal apoptosis precedes gross necrosis [21], [22]. Sites of increased epithelial apoptosis have been linked with elevated conductance of the intestinal epithelium characteristic of increased permeability of the intestinal barrier [23].

Breast milk feeding decreases NEC incidence [24]. It has been proposed that human milk may decrease the incidence of NEC by enhancing maturation of the intestinal barrier and reducing pathogenic bacteria colonization [25]–[27], thus ameliorating the proinflammatory response [28]. The components in human milk that have NEC protective effects have not been clearly delineated. Erythropoietin (Epo) is a component of human milk [29]. Epo was originally described as a promoter of erythropoiesis through prevention of apoptosis of erythroid progenitor cells [30]. Later studies found that the Epo receptor is widely expressed in many other nonhematopoietic tissues, and the anti-apoptotic effect of Epo has been shown in brain, heart and intestine [31]–[34]. Specifically, functional Epo receptors are present in fetal and postnatal small intestines [35], [36] suggesting that Epo may play a role in the development of the gastrointestinal tract. It has been reported that Epo protects the intestine against ischemia/reperfusion injury in adult rats [37], [38]. We have previously described the protective effect of Epo on NEC incidence through preservation of intestinal barrier function via an effect on tight junction protein expression [39]. However the effect of Epo on autophagy and apoptosis in immature small intestine, especially the preterm ileum is unknown.

In this study, we hypothesize that Epo decreases the incidence of NEC and down-regulates autophagy and apoptosis in the preterm intestinal epithelium. The associations of both autophagy and apoptosis with NEC development are investigated using an experimental neonatal rat NEC model. It was found that administration of Epo reduced both autophagy and apoptosis. In addition, *in vitro* experiments were performed using rat IEC-6 cells to investigate the possible mechanisms underlying down-regulation of autophagy and apoptosis by Epo.

## Materials and Methods

### Ethics Statement

This study was carried out in strict accordance with the recommendations in the Guide for the Care and Use of Laboratory Animals of the National Institutes of Health. All animal work was conducted under the animal protocol No. 71557 and was approved by the University of Chicago Institutional Animal Care and Use Committee (IACUC). Cesarean-section was performed under isoflurane anesthesia, and all efforts were made to minimize suffering. If a rat pup showed illness during the course of the study, the animal was humanely euthanized.

### Experimental Neonatal Rat NEC Model

All animal studies were reviewed and approved by the Institutional Animal Care and Use Committee. Using our previously published methods [39], neonatal rats from timed pregnant Sprague-Dawley dams were delivered by cesarean section one day before scheduled birth. Pups were fed with Esbilac puppy formula every 3 hours via an orogastric feeding catheter and were colonized with bacteria 10^7^ CFU each of *Serratia marcescens*, *Klebsiella pneumoniae*, and *Streptococci viridans* once daily in 100 µl formula via an orogastric feeding catheter. In addition, pups were stressed with hypoxia (5% oxygen and 95% nitrogen for 10 minutes) three times a day. Some pups were sacrificed at postnatal age of day 1, day 2, day 3, or when they became morbid during the experiment. All the other surviving pups were sacrificed at the end of the experiment on day 5.

The feeding volume began at 0.1 ml and was increased incrementally up to 0.25 ml. Animals were randomly divided into 2 groups with similar average weight, and fed with formula only or formula with Epo (0.1 µg/ml). Non-stressed, naturally born full term mother-fed pups (MF) were included as healthy controls.

Half of the ileum was collected and fixed in 10% buffered formalin overnight for tissue section preparation. The other half was snap frozen in liquid nitrogen and stored at −80°C for intestinal lysate preparation for immunoblotting. Hematoxylin and eosin (H&E) stained intestinal sections were assessed histologically for ileal damage by a pathologist blinded to the treatment categories using the previously published NEC scoring system with a range of 0–4 [39]. Pups with NEC score 0 were exposed to the NEC model but did not have evidence of histologic injury. Scores ≥2 were defined as NEC, with truncated and/or denuded villi.

### Immunohistochemistry (IHC)

Paraffin-embedded intestinal sections were deparaffinized at 56°C, immersed in xylene three times and hydrated with ethanol (two times with 100%, two times with 95% and one time with 75% ethanol) for 5 minutes each. For antigen unmasking, slides were heated in 10 mM sodium citrate buffer (pH 6.0) for 10 minutes prior to treatment with 3% hydrogen peroxide for 10 minutes. The specimens were treated with 5% BSA in TBST (Tris-buffered saline with 0.1% v/v Tween-20) for 1 hour at room temperature followed by overnight incubation with a rabbit polyclonal Beclin 1 antibody (ProSci, Inc., Poway, CA), rabbit polyclonal cleaved caspase-3 antibody (Cell signaling, Danvers, MA) or mouse monoclonal anti-LC3 antibody (nanoTools, Teningen, Germany) at 4°C. After washing, the sections were incubated with anti-rabbit HRP or anti-mouse HRP for 30 minutes at room temperature (Dako, Carpinteria, CA). Positive staining was visualized with DAB chromogen and nuclei counterstain was performed with hematoxylin. Images were acquired by Olympus FSX100. For quantitation of Beclin 1, LC3, and cleaved caspase-3 staining, the intensity of IHC staining was scored on a “0” to “3” scale with “0” being no staining, “1” being weak staining, “2” being moderate staining and “3” being strong staining among slides examined [40].

### Immunofluorescence (IF)

After deparaffinization and rehydration, intestinal sections were blocked in 5% normal goat serum (Sigma, St. Louis, MO) for 1 hour at room temperature and then incubated with mouse monoclonal LC3 antibody (nanoTools, Teningen, Germany) and mouse monoclonal p62 antibody (BD Biosciences, San Jose, CA) at 1∶100 dilution, followed with an Alexa Fluor 488-conjugated second antibody (Molecular Probes, Eugene, OR). Nuclei were labeled with 4′, 6-diamidino-2-phenylindole (DAPI) (100 ng/ml) (Sigma, St. Louis, MO). Coverslips were mounted on slides using SlowFade Gold anti-fade reagent (Invitrogen, Grand Island, NY). Images were acquired by Olympus DSU Spinning Disk Confocal and analyzed using Slidebook Software (Olympus Inc., Center Valley, VA).

### Immunoblotting

For tissue lysate preparation, small intestinal tissues were minced and sonicated in 300 µl RIPA buffer (50 mM Tris•HCl, pH 7.4; 150 mM NaCl; 1% Nonidet P-40 (NP-40); 0.1% sodium dodecyl sulphate (SDS); 0.5% sodium deoxycholate) containing protease inhibitors (Roche, Indianapolis, IN) and phosphatase inhibitor (Thermo Scientific, Rockford, IL). For cell culture lysate preparation, cells on plates were rinsed with cold PBS and lysed in the same lysis buffer used for tissue lysate preparation. The total protein concentration in lysates was determined using a BCA™ protein assay kit (Pierce, Rockford, IL). Lysates were resolved on SDS–PAGE. After electrophoresis, proteins were blotted onto a nitrocellulose membrane. The blots were blocked in 5% milk PBS-T (0.1% Tween 20 (v/v) in phosphate-buffered saline) for 1 hour at room temperature and then probed with primary and appropriate secondary antibodies in 5% milk PBS-T. Immunoreactive bands were visualized by chemiluminescence reaction using ECL reagents (Pierce, Rockford, IL) followed by exposure of the membranes to autoradiography film (Denville scientific, Metuchen, NJ). Protein levels were quantified by densitometry using ImageJ software. The LC3 antibody was purchased from nanoTools (Teningen, Germany), the Beclin 1 antibody was from ProSci (Poway, CA), the p62 antibody was from BD Biosciences (San Jose, CA), the phospho-Akt, phospho-mTOR, phospho-p70S6K, phospho-p44/p42 MAPK, Akt, phospho-Jak2, Jak2 and Bcl-2 antibodies were purchased from Cell signaling (Danvers, MA). The β-actin antibody was from Sigma (St. Louis, MO). Protein bands of interest were quantitated by densitometry and normalized to β-actin.

### TUNEL Staining

Formalin-fixed, paraffin-embedded intestinal tissue sections were deparaffinized, treated with proteinase K (20 µg/ml) for 15 minutes at 37°C, and washed twice in PBS for 5 minutes each. The *in situ* labeling of fragmented genomic DNA was done by using the *In Situ* Cell Death Detection Kit TMR Red (Roche, Indianapolis, IN) for 1 hour at 37°C. After labeling, preparations were washed and nuclei counter-stained with DAPI (100 ng/ml) (Sigma, St. Louis, MO). After three washes in PBS, the slides were mounted with SlowFade Gold anti-fade reagent (Invitrogen, Grand Island, NY). Processing without terminal transferase was performed as a negative control to ensure specificity of positively stained apoptotic bodies. Slides were examined with a Leica TCS SP2 Confocal Microscope. Fluorescence images were then pseudo-colored using ImageJ software and overlaid to illustrate the localization of TUNEL positive nuclei relative to total nuclei in the intestine.

### Cell Culture

The non-transformed rat intestinal epithelial cell line, IEC-6 (ATCC, Manassas, VA; CRL-1592), was maintained in DMEM containing 10% fetal bovine serum (FBS), 2% penicillin, 2% streptomycin, 4 mM L-glutamine, 0.01 mg/ml insulin (Sigma, St. Louis, MO) at 37°C, 5% CO_2_ incubator. Cell passages 25–35 were used.

### Autophagosome Formation

After different treatments for 6 hours, IEC-6 cells were washed in PBS and then fixed with 4% paraformaldehyde (Electron Microscopy Sciences, Hatfield, PA) for 15 minutes at room temperature. Fixed cells were washed with PBS, permeabilized in 100% methanol for 10 minutes at −20°C, washed in PBS, and blocked in blocking buffer (X0909; DAKO, Carpinteria, CA) for 1 hour at room temperature. Cells were subsequently incubated with anti-LC3 antibody in antibody diluent (S3022; DAKO, Carpinteria, CA) overnight at 4°C. After three TBST washes, cells were incubated with Alexa Fluor 488-conjugated secondary antibody (Invitrogen, Grand Island, NY) for 1 hour at room temperature and then washed three times in TBST. Nuclei were labeled with DAPI (100 ng/ml) (Sigma, St. Louis, MO). Coverslips were mounted on slides using SlowFade Gold anti-fade reagent (Invitrogen, Grand Island, NY). Cells were observed with an Olympus DSU Spinning Disk Confocal microscope, photographed at a magnification of ×600 and analyzed using Slidebook Software (Olympus Inc., Center Valley, VA).

### Cell Death Detection ELISA

An estimate of the extent of DNA fragmentation after apoptosis induction with or without Epo pretreatment was carried out using the cell death detection ELISA kit (Roche, Indianapolis, IN). Lysates from IEC-6 cells (1×10^4^ cells/ml) were prepared, overlaid and incubated in microtiter plate modules coated with anti-histone antibody for apoptosis detection. DNA fragmentation was estimated by spectrophotometric measurement of microtiter plates in a BioTek Elx800 plate reader at 405 nm.

### Reagents

Recombinant rat erythropoietin was purchased from R&D Systems (Minneapolis, MN). Recombinant rat TNF-α was from PeproTech (Rocky Hill, NJ). Inhibitors used to inhibit activation of intracellular pathways (AG490 for JAK2 pathway, PD 98059 for MAPK/ERK pathway and LY 294002 for the PI3K/Akt pathway) were from EMD and rapamycin for the mTOR pathway was from Enzo life sciences (Farmingdale, NY).

### Statistical Analysis

Data were expressed as means ± SD. Statistical analysis was performed using the two-tailed Student’s *t*-test for paired data. Difference was considered to be significant at *p* value <0.05.

## Results

### Increased Autophagy and Apoptosis in Experimental NEC

Autophagy and apoptosis are two interconnected pathways in response to cellular stress. Under normal physiological conditions, basal levels of autophagy and apoptosis contribute to intestinal epithelial homeostasis. Under persistent stressed conditions, however, autophagy can no longer support cell survival, instead excessive autophagy and massive activation of apoptosis lead to severe injury of intestinal mucosa and gut barrier failure. We first examined whether elevated autophagy or apoptosis were associated with NEC in our experimental model.

**Figure 1:**
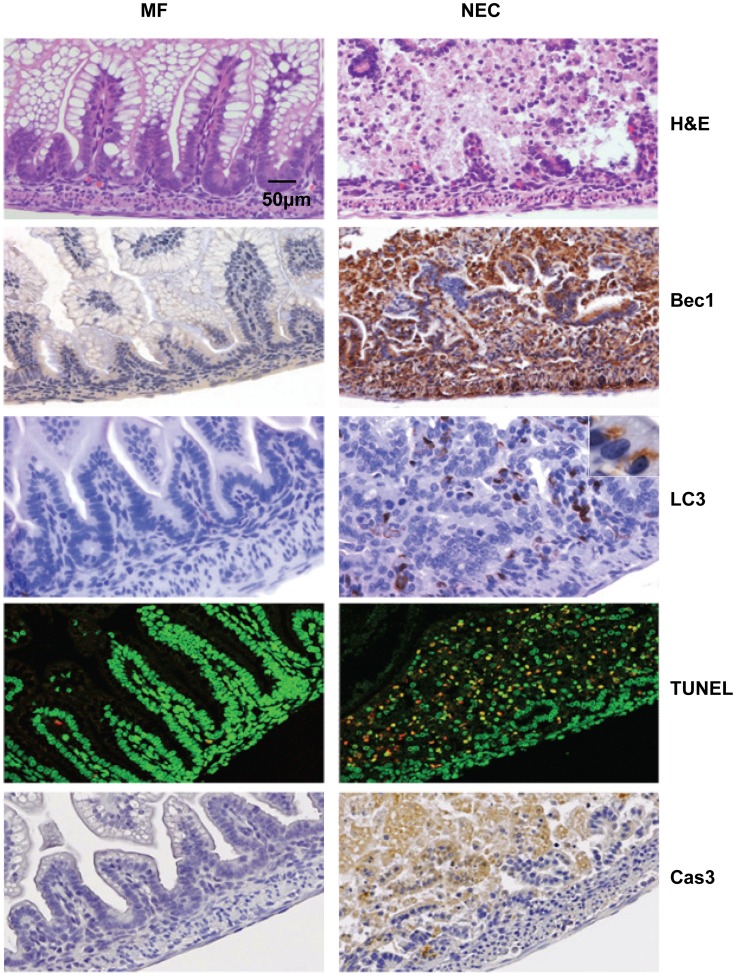
Increased autophagy and apoptosis in experimental neonatal rats with NEC. Representative slides for MF pups and NEC pups processed by H&E, Beclin 1 (Bec1), LC3, TUNEL and cleaved caspase-3 (Cas3) staining. Immunofluorescence of TUNEL positive enterocytes is shown in red and DAPI nuclear counterstaining is pseudo-colored green (TUNEL). In MF pups, the villi structure was intact (H&E, left panel); intestinal epithelial cells exhibited low basal level of Beclin 1 (Bec1, left panel) and LC3 expression (LC3, left panel); rarely TUNEL positive enterocytes (TUNEL, left panel) and almost no positive cleaved caspase-3 signal (Cas3, left panel). In NEC pups (with NEC score ≥2 and no Epo treatment), villus structure is damaged (H&E, right panel) associated with extensive Beclin 1 (Bec1, right panel) and LC3 signal (LC3, right panel) with punctuate staining of typical LC3II (indicated in the enlarged inset); TUNEL positive enterocytes (TUNEL, right panel, yellow dots) and cleaved caspase-3 positive enterocytes (Cas3, right panel) were observed. (n = 6 animals per group) Original magnification, ×20 and inset ×100. Scale bar = 50 µm.

Ileum sections from mother fed healthy control pups (MF) and experimental pups with NEC (NEC score ≥2) (NEC) were compared. To eliminate differences due to developmental stage, all sections were from the pups sacrificed on the same postnatal days. In H&E stained specimens of MF pups, the morphology of the intestinal villi was intact with no morphologic alteration (Figure 1, H&E, left); while in the specimen of NEC pups, the villi structure was disrupted (Figure 1, H&E, right). Autophagy was evaluated by Beclin 1 and LC3 expression. Beclin 1 and LC3 expression were significantly increased in ileum sections from NEC pups (NEC score≥2) (Figure 1, right) compared to MF healthy pups (Figure 1, left). Not only the total intensity of LC3 staining was much higher in NEC pups, the enlarged inset in Figure 1 (LC3) reveals punctuate staining of LC3 around the nucleus suggesting the typical LC3II staining pattern, regarded as the classic marker of autophagy [14]. Neither Beclin 1-positive staining nor LC3-positive staining was observed in intestinal tissue sections from NEC diseased pups in the absence of primary antibody indicating that the staining was not a nonspecific stain from the secondary antibody (data not shown).

TUNEL staining was used to evaluate apoptosis. TUNEL-positive enterocytes were rarely present in the ileum of MF pups (Figure 1, TUNEL, left), while extensive TUNEL positive staining was seen in intestinal sections from diseased pups (Figure 1, TUNEL, right). Cleaved caspase-3 staining was evaluated as an additional marker for apoptosis. Consistent with TUNEL staining results, cleaved caspase-3 was also extensively present in the epithelial cells of NEC pups (Figure 1, Cas3, right) while no cleaved caspase-3 positive cells were detected in the mother fed pups (Figure 1, Cas3, left). Taken together, these results demonstrate increased autophagy and apoptosis in experimental NEC.

### Elevated Autophagy Preceded Apoptosis in Neonatal Rat NEC Model

To determine whether autophagy and apoptosis work simultaneously or sequentially, the relative timing of autophagy and apoptosis was investigated in the NEC model from individual pups sacrificed at postnatal ages day 1 to day 3.

**Figure 2:**
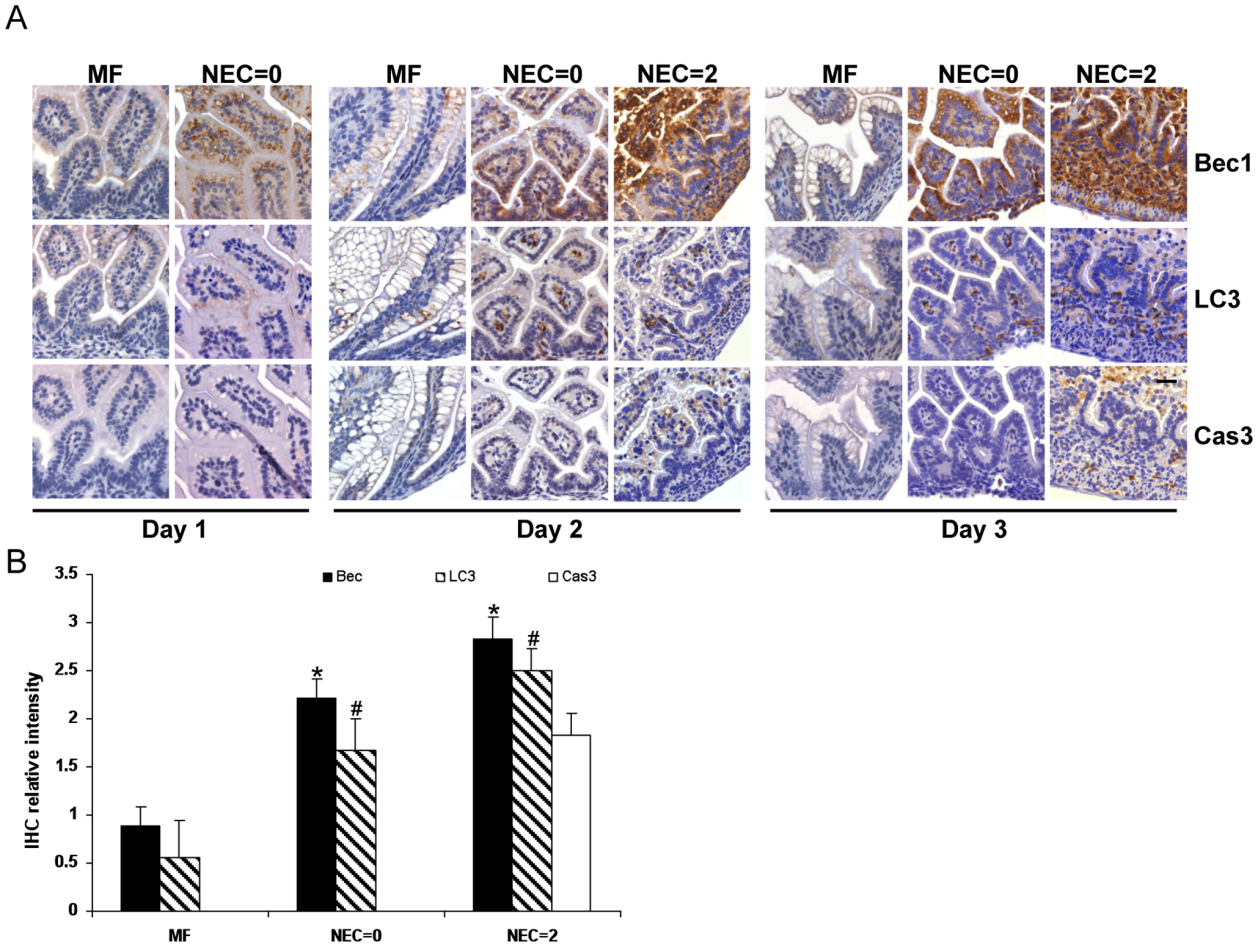
The onset of autophagy and apoptosis under experimental NEC stressed conditions. (A) Representative corresponding slides from MF and experimental NEC stressed pups stained with Beclin 1(Bec1), LC3 and cleaved caspase-3 (Cas3) from postnatal age day 1 to day 3. In MF pups (left column in each set of panels for each day), only low basal level of Bec1 and LC3 expression were observed with no positive apoptosis signal across all ages. In experimental NEC stressed pups (NEC = 0 and NEC = 2), increased intensity of Bec1 and LC3 signal was present throughout the entire ileum section. The cleaved caspase-3 (Cas3) positive signal was only observed in ileum sections with NEC score 2. (n = 3 animals for each time point) Original magnification, ×40. Scale bar = 50 µm. (B) IHC intensity of Bec1, LC3, Cas3 from A. was scored on a 0–3 scale. Results are presented as mean of scores ± SD. * and # depict p<0.05 compared to MF group by Student’s *t*-test in Bec1 intensity and LC3 intensity respectively.

In healthy MF control pups, only low basal level expression of Beclin1 and LC3 was detected, and no activation of cleaved caspase-3 was seen in any of the intestinal sections obtained from day 1 to day 3 (Figure 2A and 2B, MF). However, increased expression of the autophagy markers Beclin 1and LC3 were noted with prolonged NEC stress. On postnatal age day 1, although no pup had evidence of NEC by histology as no damage to the villi of the epithelial cells was observed, increased expression of Beclin 1 and LC3 was already detectable in pups exposed to the stresses of the experimental NEC model, indicating up-regulated autophagy. On postnatal age day 2 and 3, in pups exposed to NEC stress but without evidence of disease by histologic NEC scoring (NEC = 0), there was elevated expression of Beclin 1 and LC3 without evidence of cleaved caspase-3 (Figure 2A and 2B, NEC = 0). In pups with NEC disease as evidenced by the damage of villi structure with intestinal histologic scoring 2, Beclin 1, LC3 and cleaved caspase-3 were all significantly increased (Figure 2A and 2B, NEC = 2). These data suggest that autophagy precedes the onset of apoptosis in the experimental rat NEC model. Autophagy and apoptosis only co-existed in the more severely injured rat intestine of pups diagnosed with NEC.

### Epo Treatment Reduced Autophagy in Experimental Neonatal Rat Small Intestine

**Figure 3:**
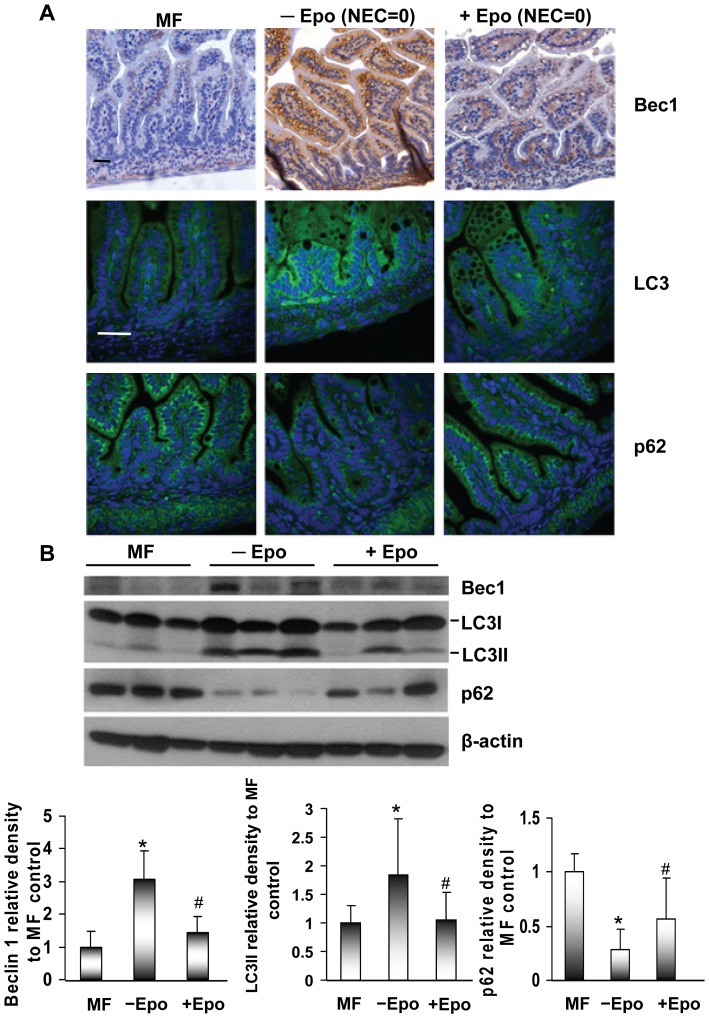
Effect of Epo on Beclin 1, LC3 and p62 expression in ileum of experimental NEC rats. (A) IHC staining of Beclin 1 (Bec1), IF staining of LC3 and p62 in ileum sections of MF pups and experimental pups without or with Epo administration (–Epo vs. +Epo). Ileum sections were from the pups sacrificed at the end of the experiment day (day 5) with NEC score 0. The sections from age-matched MF pups were added as healthy control. (n = 9 animals for each group) Original magnification, ×20 for IHC, ×40 for IF. Scale bar = 50 µm. (B) Representative western blots of Beclin 1 (Bec1), LC3, p62 expression in intestinal lysates from MF control pups (MF) and the experimental pups without or with Epo administration (–Epo vs. +Epo), n = 9 animals for each treatment group. All of the lysates were from the pups sacrificed at the end of the experiment day (day 5) with NEC score 0. Significantly decreased Beclin 1, LC3II isoform and increased p62 expression was detected in the intestinal lysates of experimental pups with Epo treatment compared to pups without Epo treatment. The ratios of Beclin 1, LC3-II, p62 density to β-actin were calculated respectively using ImageJ software, set to one for MF control pups. Data are presented as means ± SD. * indicates *p*<0.05 non-Epo treated group compared with MF group, # indicates *p*<0.05 Epo treated group compared with non-Epo treated group by Student’s *t*-test. Images are representative 3 separate experiments.

To investigate whether the protective effect of Epo was associated with autophagy, the ileum sections from the pups that survived to day 5 of the experiment with NEC score 0 (exposed to NEC stress for 5 days, but no evidence of disease) were examined. These pups were chosen to exclude the confounder of intestinal necrosis associated with disease state. Age-matched MF pups were used as healthy controls. The overall IHC staining of Beclin 1 was decreased in ileum from Epo treated pups (+Epo) compared to sections from non-Epo treated pups (−Epo), close to the low expression levels in MF pups (Figure 3A, upper panel). In order to get more accurate information of LC3 and p62 expression among different groups, IF staining instead of IHC was applied with mouse monoclonal LC3 and p62 antibodies. Correspondingly, the strong staining of LC3 was detected in non-Epo treated pups (−Epo). MF pups and Epo treated pups (+Epo) had comparatively low LC3 expression (Figure 3A, middle panel). p62 is a substrate of autophagy. It incorporates into autophagosomes through direct binding to LC3 and is efficiently degraded by autophagy. Thus the total expression level of p62 is inversely correlated with autophagic activity. The opposite expression pattern of p62 to Beclin 1 and LC3 among different groups further verified the occurrence of increased autophagy activation in the non-Epo treated group (Figure 3A, lower panel).

The protein expression level of Beclin 1, LC3I/II and p62 was also analyzed in small intestine lysate harvested from surviving pups at the end of NEC experiments with NEC score 0. The significant decrease of Beclin 1, LC3II isoform, and marked increase of p62 in Epo treated pups compared to non-Epo treated pups clearly demonstrates that Epo treatment can reduce autophagy in early NEC development (Figure 3B).

### Epo Treatment Reduced Apoptosis and Up Regulated Bcl-2 Expression in Experimental Neonatal Rat Small Intestine

**Figure 4:**
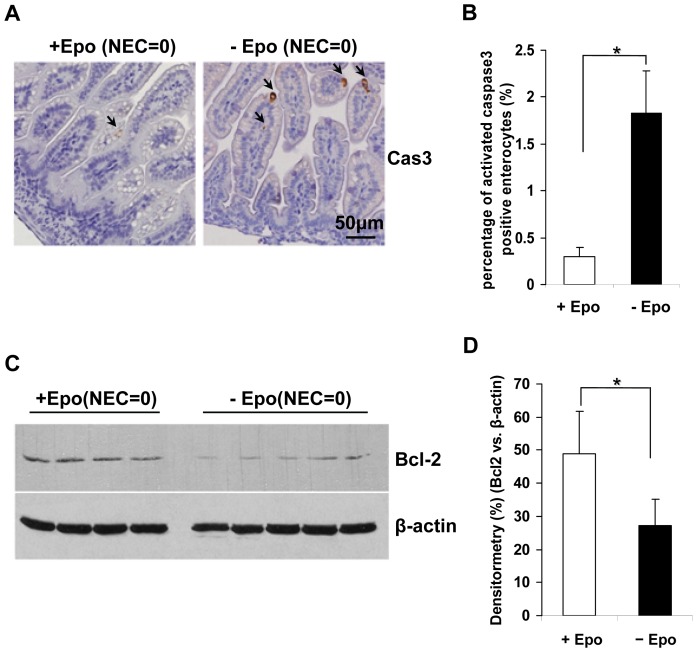
Epo treatment reduced apoptosis and up regulated Bcl-2 expression in experimental neonatal rat ileum. (A) Cleaved caspase-3 staining of ileum section with NEC score 0 as described in Materials and Methods, with or without Epo administration (+Epo vs. –Epo). Original magnification, ×40. Scale bar = 50 µm. (B) The percentage of positive cleaved caspase-3 enterocytes per HPF. Data are shown as the mean ± SD obtained from 3 separate experiments with minimum 10 HPFs. * indicate *p*<0.01 by Student’s *t*-test. (C) Corresponding intestinal lysates from the same set of pups were collected and subjected to immunoblotting for Bcl-2 and β-actin. Representative immunoblots from three experiments with similar results are shown (n = 10 animals for each treatment group). (D) Densitometric values were normalized to β-actin. * depicts *p*<0.05 by Student’s *t*-test.

As the data indicated that NEC stress-induced autophagy preceded the onset of apoptosis, the effect of Epo treatment on apoptosis was next tested. As above, in order to remove the confounder of apoptosis associated with necrosis and to eliminate differences due to NEC development stage among the pups, only ileum sections from the surviving pups at the end of experiment with NEC score 0 were examined. Since there was almost no positive cleaved caspase-3 signal detected in MF ileum specimens, cleaved caspase-3 was only evaluated in Epo treated and untreated rat ileum by IHC staining. Compared to NEC diseased pups (Figure 1, Cas3, right), the number of total cleaved caspase-3 positive ileal epithelial cells was significantly decreased in experimental pups with NEC score 0 (Figure 4A). Furthermore, there was a significant difference in the percentage of cleaved caspase-3 positive enterocytes between the two groups at NEC stage “0″ with or without Epo treatment. Epo treated pups had significantly reduced cleaved caspase-3 signal compared to untreated pups. (Figure 4A and 4B, +Epo vs.-Epo, *p*<0.01).

The corresponding small intestine lysates from these pups were further tested for the expression of the anti-apoptotic protein Bcl-2. Bcl-2 was significantly increased in pups with Epo administration compared to pups without Epo treatment (Figure 4C and 4D, *p*<0.05), indicating that Epo may reduce caspase-3 activation via up regulation of the anti-apoptotic protein Bcl-2.

### Epo Treatment Reduced Autophagy in Serum Deprived IEC-6 Cells

The regulatory mechanism of Epo reduced autophagy was further investigated in the nontransformed rat small intestinal epithelial cell line, IEC-6. Autophagy was induced in IEC-6 cells by serum starvation for different time periods-3, 6, 12, 24 hours. The expression level of the autophagy marker LC3II was evaluated by western blotting. The peak expression of LC3II was found at 6 hours of serum withdrawal (data not shown), therefore, 6 hours serum starvation was applied for all the subsequent studies. Under conditions of nutrient excess, mTOR is a key negative regulator of autophagy. Inhibition of mTOR with specific inhibitors such as rapamycin induces activation of multiple ATG proteins which leads to autophagosome formation [12]. Thus rapamycin was used as a positive autophagy activation control. To determine the optimal dosage of Epo for autophagy reduction, cells were treated with various concentrations of Epo ranging from a low dosage at 0.6 U/ml to a high dosage at 60 U/ml [36].

**Figure 5:**
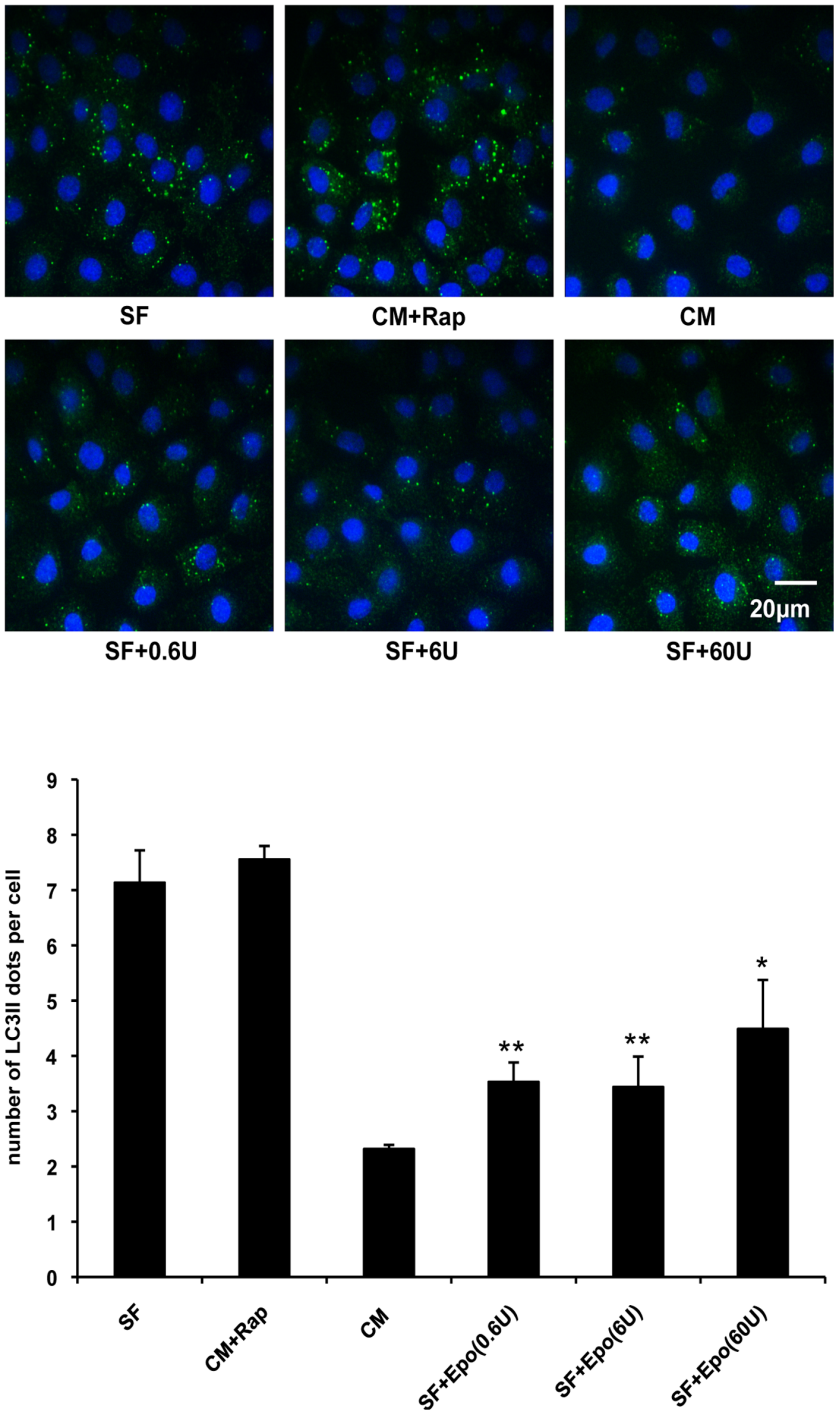
Epo treatment reduces autophagy in serum deprived IEC-6 cells. IEC-6 cells were incubated in serum free media (SF), complete media+rapamycin (CM+Rap, Rap: 100 nmol/l), complete media (CM) and serum free media+Epo (0.6 U/ml, 6 U/ml, 60 U/ml) (SF+0.6U, SF+6U, SF+60U) for 6 hours. After treatment, IEC-6 cells were fixed and stained for LC3 (green dots), nuclei were stained with DAPI (blue). Autophagosome formation in IEC-6 cells as reflected by LC3II puncta was observed using confocal microscopy. Images are representative of 3 separate experiments. The number of LC3II puncta was quantified and counted using ImageJ software in minimum 300 cells for each condition. Data are presented as means ± SD. * and ** indicate *p*<0.05 and *p*<0.01 respectively, compared with serum starved cells by Student’s *t*-test. Original magnification, ×600. Scale bar = 20 µm.

Autophagy was determined by autophagosomes indicated by the amount of the punctate form of LC3 (green dots in Figure 5). The number of green dots representing LC3II, was dramatically increased in serum deprived cells (Figure 5, SF) or cells in complete media treated with rapamycin (Figure 5, CM+Rap), compared to control cells without any treatment in complete media (Figure 5, CM). Administration of various concentrations of Epo (0.6 U/ml to 60 U/ml) (Figure 5, lower panels) to serum free cells significantly decreased the abundance of LC3II dots compared to non-Epo treated cells, (Figure 5, SF).

### Akt/mTOR Pathway is Involved in Epo Regulated Autophagy

**Figure 6:**
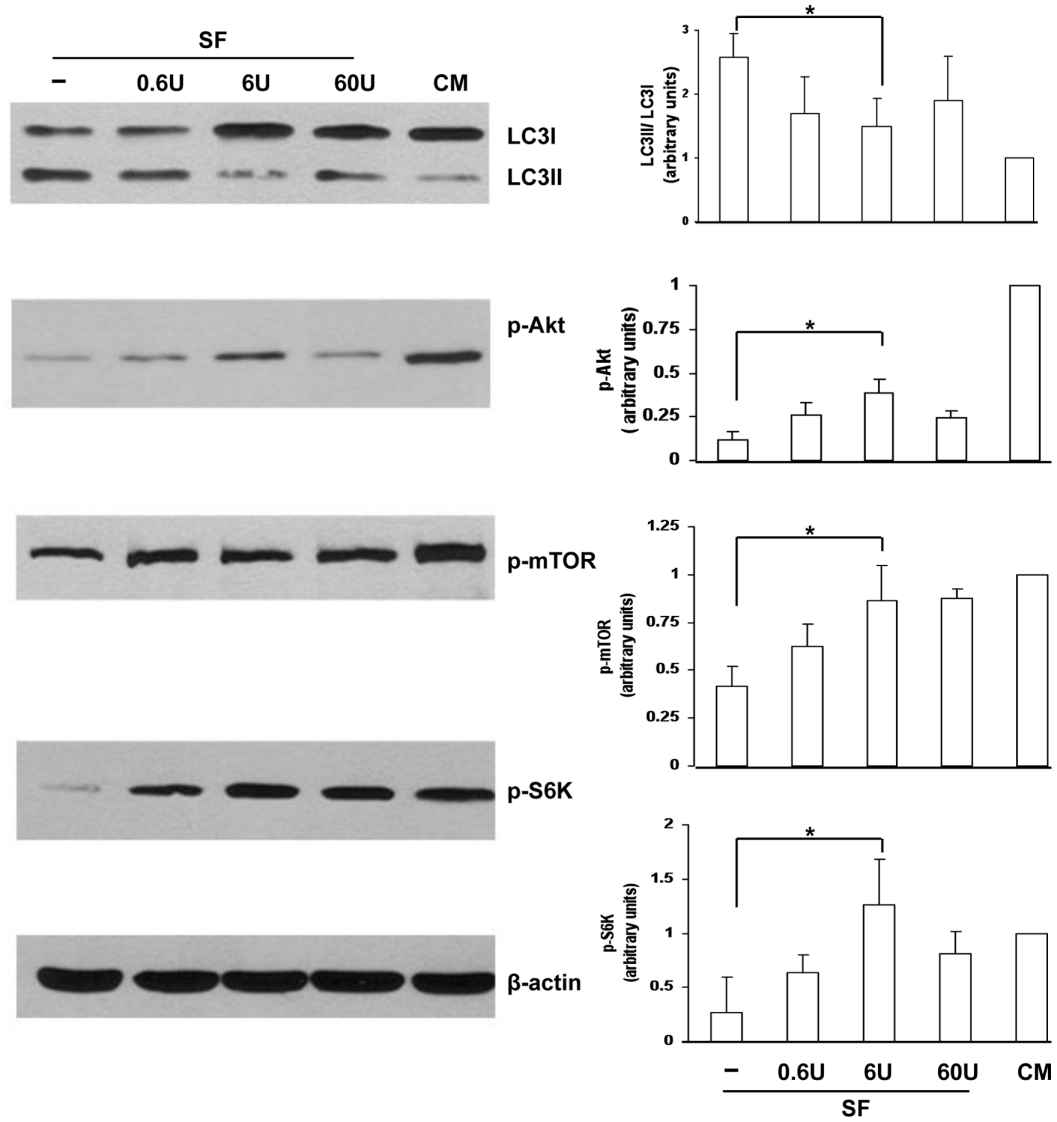
Akt/mTOR pathway is involved in Epo regulated autophagy in IEC-6 cells. IEC-6 cells were incubated in serum free media (**–**) or serum free media+Epo (0.6 U/ml, 6 U/ml, 60 U/ml) for 6 hours. Cell culture with complete media (CM) was included as treatment control. The lysates of IEC-6 cells were collected and analyzed by western blotting for LC3, p-Akt, p-mTOR, p-S6K. Densitometric values were obtained using ImageJ software and normalized to β-actin, set to one for cells with complete media (CM). Data are presented as means ± SD. * indicates *p*<0.05 compared with serum starved cells by Student’s *t*-test. Images are representative 3 separate experiments.

The Akt/mTOR signaling pathway plays an important role in autophagy regulation during growth factor and nutrient deprivation [41]. In Akt/mTOR signaling, activated Akt phosphorylates and decreases the ability of tuberous sclerosis complex 2 (TSC2) to inhibit mTOR, resulting in activation of mTOR and its downstream target p70S6K (S6K) [42], [43]. Thus whether Epo regulates autophagy via activation of Akt/mTOR signaling was next investigated. Figure 6 shows that the LC3II/LC3I ratio was significantly reduced in serum free cells supplemented with 6 U/ml Epo, indicating decreased autophagy. This was associated with significant restoration of phosphorylated Akt (p-Akt) expression and upregulation of phosphorylated mTOR (p-mTOR) and phosphorylated p70S6K (p-S6K).

### MAPK/ERK Pathway is Involved in Epo-regulated Anti-apoptotic Protein Expression

The anti-apoptotic effect of Epo and its regulatory mechanism were also examined in IEC-6 cells. TNF-α plays a crucial role in the pathogenesis of NEC and other IBDs [44], [45]. In addition to acting as an inflammatory mediator, TNF-α has also been associated with the alteration of epithelial junctions and induction of apoptosis in intestinal epithelial cells [45]–[47]. However, in IEC-6 cells, TNF-α alone is not sufficient to induce apoptosis. Suppression of synthesis of short-lived anti-apoptotic proteins by cycloheximide (CHX) is required for TNF-α induced apoptosis [48]. Therefore, TNF-α (50 ng/ml) plus CHX (2.5 µg/ml) was used to examine apoptotic signaling in intestinal epithelial cells.

**Figure 7:**
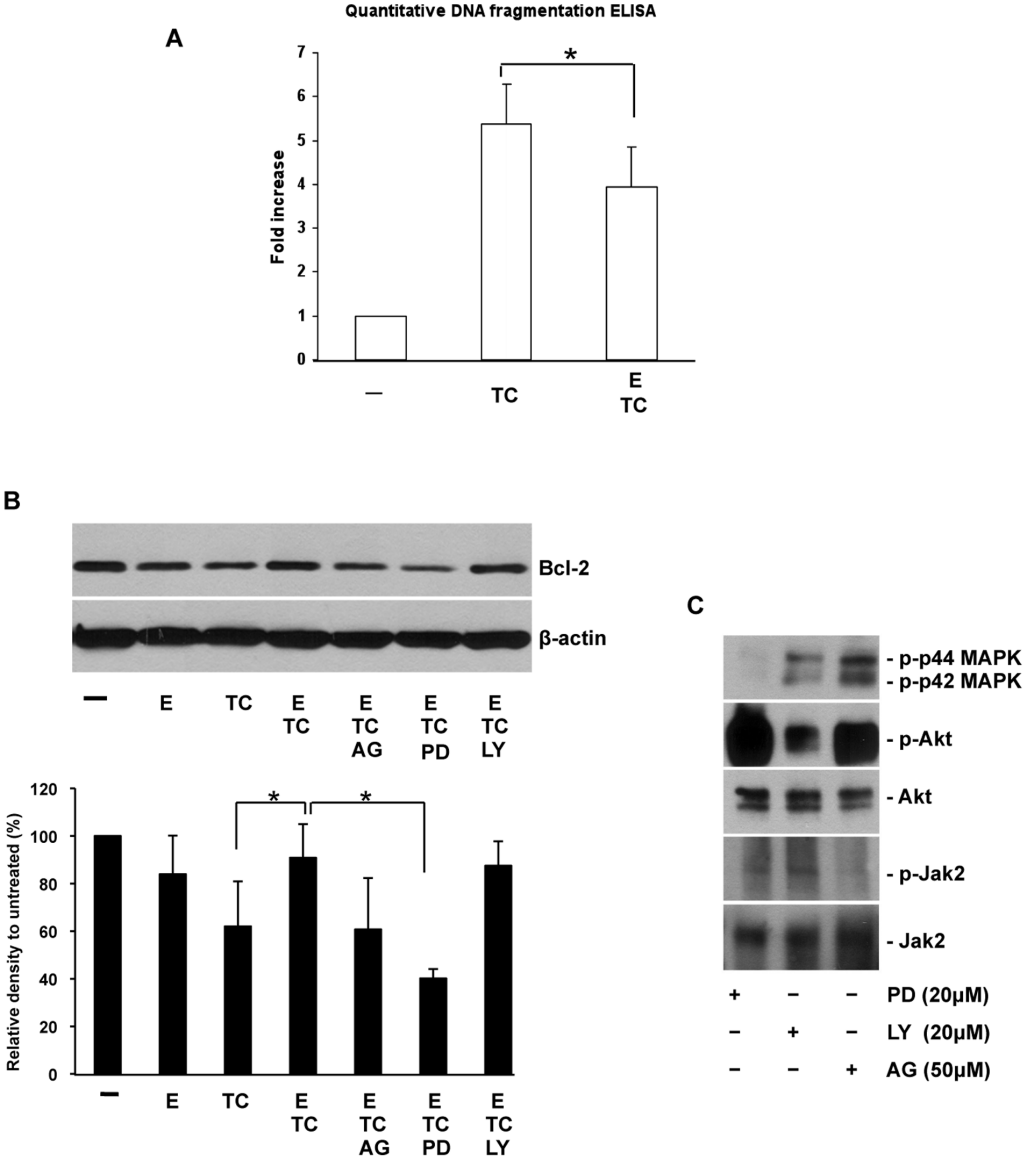
Epo reduces apoptosis in IEC-6 cells by up regulating Bcl-2 through the MAPK/ERK pathway. Apoptosis in IEC-6 cells was induced by TNF-α**/**CHX for 6 hours. IEC-6 cells were either pre-incubated with 6 U/ml Epo for 24 hours (ETC) or without Epo pretreatment (TC). Control cells received no treatment (−). (A) Cells were lysed and assayed for DNA fragmentation at the end of the treatment. DNA fragmentation was measured by a cell death detection ELISA kit. Results are presented as the fold increase in treatment group over control group (−) and represent values of three independent experiments. 24 h pretreatment with Epo decreased TNF-α/CHX induced apoptosis in IEC-6 cells. (B) Assays in the presence of the inhibitors, AG490 (50 µM) (ETCAG) or PD98059 (20 µM) (ETCPD) or LY294002 (20 µM) (ETCLY) were carried out 15 minutes before the addition of Epo. The expression of Bcl-2 was detected by western blotting. Densitometric values were normalized to β-actin. Typical images from at least three independent experiments are shown. * depicts *p*<0.05 by Student’s *t*-test. (C) The inhibitory effects of PD98059, LY294002 and AG490 were confirmed by western blotting analysis of phospho-p44/42 MAPK (p-p44 MAPK/p-p42MAPK), phospho-Akt (p-Akt) and total Akt (Akt), phospho-Jak2 (p-Jak2) and total Jak2 (Jak2) in IEC-6 cells after 15 minutes treatment with PD98059 (20 µM); LY294002 (20 µM) or AG490 (50 µM) respectively.

IEC-6 cells were pretreated with Epo for 24 hours at a dose of 6 U/ml based on a previously published study and the results of our dose response experiments [36]. Apoptosis was induced by TNF-α/CHX for another 6 hours. Apoptosis was measured by a quantitative DNA fragmentation ELISA. As shown in Figure 7A, a significant increase in DNA fragmentation was induced by TNF-α/CHX, and Epo pretreatment can decrease this apoptosis induction (*p*<0.05).

Consistent with the *in vivo* data, Epo inhibition of apoptosis was associated with up regulation of Bcl-2 protein expression (Figure 7B, ETC vs. TC). The major three intracellular signaling cascades downstream of Epo receptor are: janus tyrosine kinase 2 (JAK2); phosphatidylinositol-3 kinase (PI3K)/Akt and mitogen-activated protein kinase (MAPK)/ERK. To elucidate the signaling pathway involved in the anti-apoptosis effect of Epo in intestinal epithelial cells, specific inhibitors of these three major pathways were applied (AG490 for JAK2, PD 98059 for MAPK and LY294002 for PI3K). Significant inhibition of Bcl-2 expression was observed with inhibition of the MAPK/ERK pathway by PD98059 (*p*<0.05). In the presence of AG490 (JAK2 inhibitor) and LY294002 (PI3K inhibitor), there was no significant inhibition of Bcl-2 expression. The dosage of each inhibitor was confirmed by testing the efficiency of its inhibitory activity (Figure 7C).

## Discussion

In the present study, we find that autophagy and apoptosis are both up regulated in experimental NEC. Furthermore, autophagy precedes apoptosis in intestinal injury in NEC. Enteral supplementation of Epo can significantly down-regulate both autophagy and apoptosis, which correlates with our previously published findings of improved barrier function and lowered NEC incidence with enteral Epo treatment [39].

Increased intestinal permeability in the premature gut is an early event in NEC pathogenesis [2], [25], [39]. Although the causes of this initial epithelial injury remain unclear, primary apoptotic and autophagic mechanisms have been invoked [9], [10], [16], [21]. It has been shown in a rat NEC model that apoptosis precedes gross intestinal tissue necrosis [21]. Excessive nitric oxide production [49], exaggerated TLR4 signaling [22], and deficiency of epithelial trophic factors such as epidermal growth factor [50] have all associated with the induction of apoptosis. Our data further demonstrate that autophagy and apoptosis are both rapidly up regulated in experimental NEC *in vivo* and that autophagy precedes the onset of apoptosis in experimental NEC. However, our data is not sufficient to establish a causal relationship between autophagy or apoptosis and NEC.

Autophagy is less studied in NEC compared to apoptosis. Recently, studies have attempted to understand the regulatory role of autophagy in intestinal development and autophagy-related intestinal disease [51]. However, whether autophagy plays a role in NEC pathogenesis is largely unknown. Additionally, although increased autophagy has been observed in human NEC and experimental NEC in animals [9], [10], the connection between apoptosis and autophagy has not yet been described in NEC. In our rat experimental NEC model, basal levels of autophagy were reflected by faint Beclin 1 staining in the villi of healthy mother fed pups (Figure 1, left, Bec1). Similarly, in cultured IEC-6 cells with complete cell culture media, basal levels of autophagosome formation (Figure 5, CM) and expression of the LC3II isoform were also detected (Figure 6), suggesting autophagy is necessary for homeostasis. Under NEC stressed conditions in our model, autophagy was induced dramatically prior to apoptosis or morphologic evidence of intestinal injury (Figure 2). Increased severity of intestinal injury was associated with increased autophagy. Uncontrolled autophagy may destroy critical cellular constituents due to excessive degradation, and ultimately lead to cell death. These findings led to our further study of the regulation of autophagy and apoptosis in early NEC.

Breast milk feeding protects preterm infants from NEC development [52]. There are many potential beneficial factors in breast milk including SIgA (and SIgM), immune cells, cytokines, and growth factors [53]. Among these is Epo. The presence of a functional Epo receptor in fetal and postnatal small intestine of human and rats suggests that Epo may play a role in intestinal development. We have previously demonstrated that Epo can protect intestinal epithelial barrier function and lower the incidence of NEC through maintaining tight junction protein expression [39]. In the present study, we further show the additional protective functions of Epo in dual roles of down regulating both autophagy and apoptosis in a rat experimental NEC model. We demonstrate that under stressed conditions in a rat experimental NEC model, oral administration of Epo inhibits ileal autophagy as indicated by the decreased expression of Beclin 1, LC3II and increased expression of p62 compared to non-Epo treated pups (Figure 3). Consistently, in intestinal epithelial IEC-6 cells, Epo supplementation in serum free media significantly reduced autophagosome formation, possibly through the Akt/mTOR pathway. The Akt/mTOR pathway is one of the key pathways which regulates autophagy in the gut by sensing nutrient levels [54]. mTOR is the catalytic subunit of two distinct complexes, mTOR complex 1 and 2 (mTORC1 and mTORC2). A major signaling cascade controlling mTORC1 is the PI3K/Akt pathway [43]. After binding of growth factors to cell surface receptors, the PI3K/Akt pathway is activated and further triggers mTORC1 activation [42]. Activated mTORC1 can further activate ribosomal p70S6 kinase (S6K) and inactivate eukaryotic initiation factor 4E binding protein 1 (4EBP1), and thereby stimulate protein synthesis, cell growth and cell proliferation [55]. Our results show Epo supplementation in serum deprived cell culture media can activate Akt, which results in the cascade activation of its downstream targets, mTOR and p70S6K.

The anti-apoptotic effect of Epo in rat small intestine has been reported before. However, in this previous study, Epo was administered at high-dose via a single subcutaneous injection [56]. As the Epo receptor is expressed in multiple tissues, systemic administration has the potential risk of systemic effects. In the present study, Epo was administered enterally for a safer and more direct intestinal delivery. We found a dose response to increasing doses of Epo up to 6 U/ml, however the super-physiologic dose of 60 U/ml had a reduced effect. This may be due to saturation of the Epo receptor or receptor desensitization. It has been shown that Epo inhibits apoptosis of red blood cells by up-regulating the expression of anti-apoptotic proteins [57]. Bcl-2 is a well-known anti-apoptotic protein that can suppress apoptosis by blocking the release of cytochrome c from mitochondria and countering the effects of pro-apoptotic proteins [58]. Our study provides evidence that Epo represses apoptosis in intestinal cells through increasing the expression of Bcl-2 both *in vitro* and *in vivo*. The precise molecular mechanism underlying the up regulation of anti-apoptotic protein expression by Epo remains unclear. Several transduction cascades are activated after Epo binds to its receptor including JAK2, PI3K/Akt, MAPK/ERK pathways. Each has been indicated as essential to anti-apoptotic protein up-regulation by Epo [59]–[61]. In IEC-6 cells, we specifically found that the MAPK/ERK pathway inhibitor PD98095 blocked Epo up-regulated Bcl-2 expression. The presence of a JAK2 inhibitor or PI3K/AKT inhibitor had no effect on Epo mediated anti-apoptosis. Interestingly, the reduced apoptosis with up regulation of the anti-apoptotic protein Bcl-2 was concurrent with the reduced autophagy in the Epo treated pups in our rat NEC model. Different means of crosstalk between autophagy and apoptosis have been revealed recently: including shared signaling pathways which are induced by common stressors, such as reactive oxygen species [62]; regulation of autophagy protein by caspase-mediated cleavage [18]; regulation of apoptosis protein by autophagic flux or autophagosome formation; and direct protein-protein interactions between autophagy and apoptosis proteins. The best known example is the interaction of Bcl-2 and Beclin 1. The association of Bcl-2 with Beclin 1 is essential to antagonize Beclin 1 mediated autophagy, meanwhile Bcl-2 still retains its full anti-apoptotic activity [63]. The elegant regulation of crosstalk between autophagy and apoptosis by Bcl-2 is achieved by compartmentalization of different cellular pools of Bcl-2 in endoplasmic reticulum (ER) and mitochondria respectively with localized interacting proteins [18], [64]–[66]. Further studies to examine the local interaction of these proteins in different cellular organelles are needed to reveal a comprehensive role of Epo in the crosstalk between apoptosis and autophagy at the molecular level. Additional experiments are needed to further determine the pathogenic roles of autophagy and apoptosis in NEC and the pathophysiologic relevance of this crosstalk *in vivo*.

Taken together, our study reveals the following insights: 1. autophagy and apoptosis are both rapidly up regulated and autophagy precedes the onset of apoptosis in experimental NEC *in vivo*; 2. Epo protects intestinal epithelium from excessive autophagy and apoptosis suggesting enteral administration of Epo may be a potential therapeutic approach to regulate homeostasis of intestinal epithelial cells under conditions of gut stress.
